# Supplementary material for: Prediction of Dielectric Constant in Series of Polymers by Quantitative Structure-Property Relationship (QSPR)
Source: Polymers (Basel). 2024 Sep 26;16(19):2731. doi: 10.3390/polym16192731 (PMC11478900; doi:10.3390/polym16192731)

Prediction of Dielectric Constant in Series of Polymers by Quantitative Structure-Property Relationship (QSPR)

Estefania Ascencio[1,2,3] Shan He [1,2,3], Amirreza Daghighi [1,4], Kweeni Iduoku[1,4], Gerardo M. Casanola-Martin[1] Sonia Arrasate[2], Humberto González-Díaz [2,5], and Bakhtiyor Rasulev[1,4]*

1 Department of Coatings and Polymeric Materials, North Dakota State University, Fargo, ND 58102, USA
2 Department of Organic and Inorganic Chemistry, Faculty of Science and Technology, University of The Basque Country (UPVEHU), P.O. Box 644, 48080,
3 IKERDATA S.L., ZITEK, University of The Basque Country (UPVEHU), Rectorate Build ing, 48940 Leioa, Spain.
4 Biomedical Engineering Program, North Dakota State University, Fargo, ND 58105, USA.
5 IKERBASQUE, Basque Foundation for Science, 48011 Bilbao, Biscay, Spain.

*Correspondence: bakhtiyor.rasulev@ndsu.edu (Bakhtiyor Rasulev).

Supplementary Material

Table S1. Experimental data representing the dielectric constant of the polymers used in the experiments.

Number	Name	dielectric permittivity	Status
1	Poly (Fumaronitrile)	8.50	training
2	Poly (vinyl Fluoride)	8.50	training
3	Poly (vinylidene fluoride)	8.40	training
4	Poly (methyl cellulose)	6.80	prediction
5	Nylon 3	4.70	prediction
6	Poly(2-vinylpyridine)	4.64	training
7	Poly(acrylonitrile)	4.00	training
8	Poly (diallyl phenyl phosphonate)	3.84	training
9	Poly [4,4'-sulfone diphenoxy di(4- phenylene) sulfone]	3.80	training
10	nylon 12	3.60	training
11	Poly (diallyl phthalate)	3.57	training
12	nylon 6	3.50	training
13	Poly (hexamethylene adipamide)	3.50	training
14	Poly[N,N'-(p,p'-oxydiphenylene)pyromellitimide]	3.50	training
15	Poly [4,4'-diphenoxy di(4-phenylene) sulfone]	3.44	prediction
16	methyl a-chloroacrylate	3.40	training
17	Poly(amide-imide)	3.32	prediction
18	Poly(carbonate)	3.32	training
19	Poly[2,2'-(m-phenylene)-5,5'-bibenzimidazole]	3.30	training
20	nylon 11	3.30	training
21	poly(bismaleimide)	3.29	prediction
22	Poly(p-hydroxybenzoate)	3.28	prediction

23	Poly (ethylene terephthalate)	3.25	training
24	Poly (vinyl acetate)	3.25	training
25	Poly (ether ether ketone)	3.20	training
26	Poly (hexamethylene sebacamide)	3.20	training
27	Poly (cellulose propionate)	3.20	training
28	Poly [4,4'-isopropylidene diphenoxy di(4-phenylene) sulfone]	3.18	training
29	Poly (vinyl formal)	3.16	prediction
30	Ultem 1000	3.15	training
31	Poly (methyl methacrylate)	3.10	training
32	Poly (tetramethylene terephthalate)	3.10	training
33	Poly [thio(p phenylene)]	3.10	training
34	Poly (ethyl a-chloroacrylate)	3.10	training
35	Poly(oxymethylene)	3.10	training
36	Poly (p-methoxy-o-chloro styrene)	3.08	training
37	Poly (ethyl methacrylate)	3.00	training
38	Poly(oxy-2,2-dichloromethyltrimethylene)	3.00	prediction
39	Poly (1,4-cyclohexylidene dimethylene terephthalate)	3.00	training
40	Poly (chloro p-xylylene)	2.95	prediction
41	Poly (vinyl chloride)	2.95	training
42	Poly (3,4 dichlorostyrene)	2.94	prediction
43	Poly (Bisphenol A carbonate)	2.90	training
44	Poly (N vinyl carbazole)	2.90	training
45	Poly [1,1-ethane bis(4-phenyl)carbonate]	2.90	training
46	Poly (arylene ether)	2.90	training
47	Poly (vinylidene chloride)	2.85	training
48	Poly (n-butyl methacrylate)	2.82	training
49	Poly (dichloro p xylylene)	2.82	training
50	poly(benzimidazole)	2.81	training
51	Poly (m-chloro styrene)	2.80	prediction
52	Poly[oxy(2,6-diphenyl-1,4-phenylene)]	2.80	prediction
53	Poly (phenyl quinoxaline)	2.80	training
54	Poly(quinoline)	2.80	prediction
55	Poly (dimethyl siloxane)	2.75	training
56	Poly (isobutyl methacrylate)	2.70	training
57	Poly (ethyl cellulose)	2.70	training
58	Poly (vinyl butyral)	2.69	training
59	Poly(p-xylylene)	2.65	training
60	Poly (p-chloro styrene)	2.65	training
61	Poly(2,5-dichlorostyrene)	2.61	training

62	Poly[oxy(2,6-dimethyl-1,4-phenylene)]	2.60	training
63	Poly(chlorotrifluoroethylene)	2.60	training
64	Poly(a-vinyl_naphthalene)	2.60	training
65	Poly [1,1-cyclohexane bis(4-phenyl) carbonate]	2.60	training
66	Poly (vinyl toluene)	2.59	prediction
67	Poly (cyclohexyl methacrylate)	2.58	training
68	Poly (a methyl styrene)	2.57	training
69	Poly(styrene)	2.55	training
70	Poly (1,4 butadiene)	2.51	training
71	Poly (b-vinyl naphthalene)	2.51	training
72	Poly (o methyl styrene)	2.49	training
73	Poly (methyl p xylylene)	2.48	training
74	Poly(norbornene)	2.40	training
75	Poly(isoprene)	2.37	training
76	Poly(cis-1,4-Polyisoprene)	2.37	training
77	Poly (a,a,a',a' tetrafluoro-p-xylylene)	2.35	training
78	Poly(ethylene)	2.30	prediction
79	Poly(1-butene)	2.27	prediction
80	Poly (vinyl cyclohexane)	2.25	training
81	Poly(isobutylene)	2.23	prediction
82	Poly(methylpentene)	2.21	training
83	Poly(propylene)	2.20	training
84	Poly(naphthalene)	2.20	training
85	Poly(4-methyl-1-pentene)	2.13	training
86	Poly(tetrafluoroethylene)	1.85	training

Figure S1. Machine Learning prediction of polymers at different frequencies.

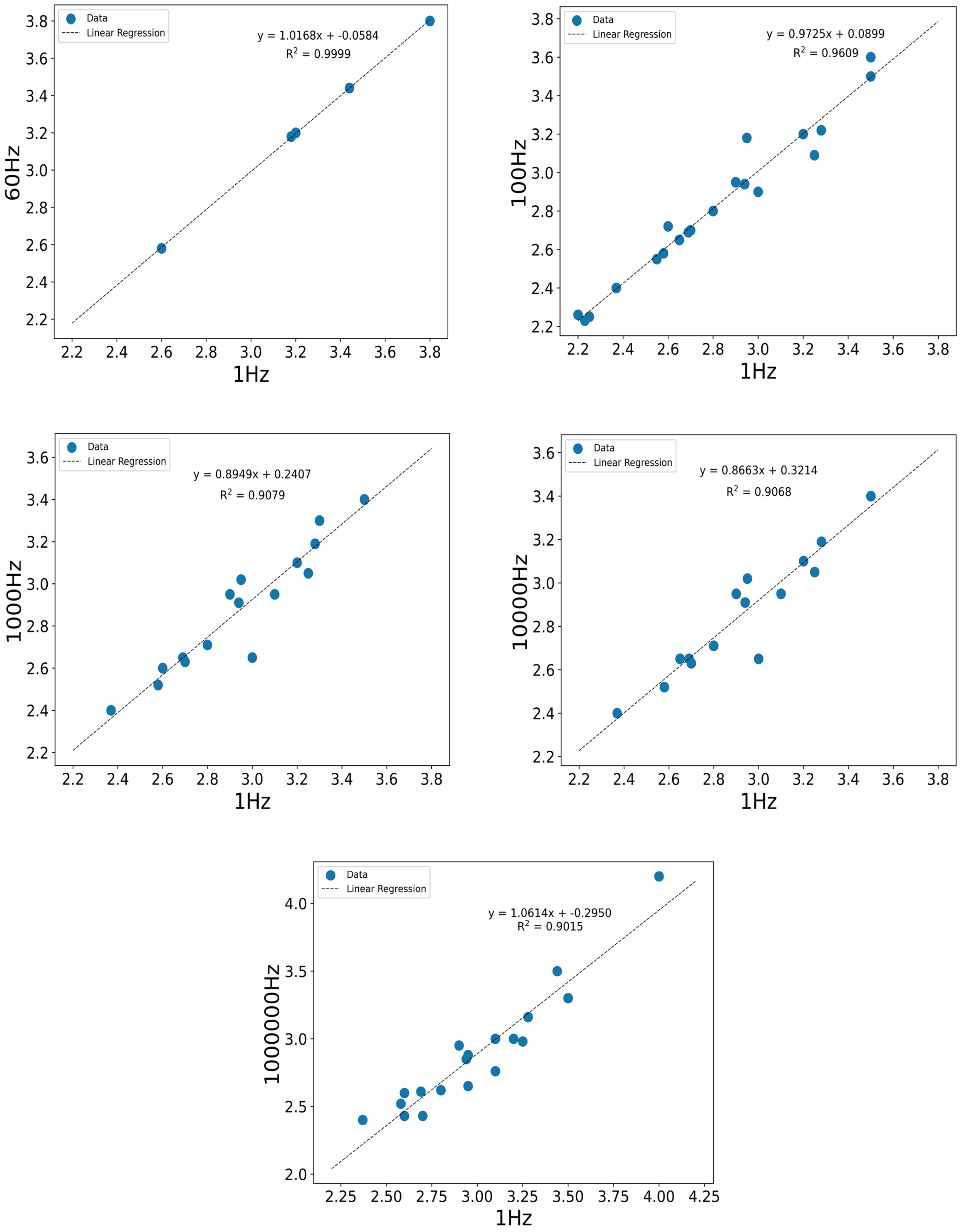

Supplement: Supplementary file 1 [file polymers-16-02731-s001.zip › polymers-3178438-supplementary.pdf]
